# Birth of spinal muscular atrophy unaffected baby from genetically at-risk parents following a pre-implantation genetic screening: A case report

**DOI:** 10.18502/ijrm.v20i9.12068

**Published:** 2022-10-10

**Authors:** Arie Adrainus Polim, Nining Handayani, Dian Kesumapramudya Nurputra, Anggia Melanie Lubis, Batara Sirait, Dennis Jakobus, Arief Boediono, Ivan Sini

**Affiliations:** ^1^Morula IVF Jakarta Clinic, Jakarta, Indonesia.; ^2^IRSI Research and Training Centre, Jakarta, Indonesia.; ^3^Department of Obstetrics and Gynecology, School of Medicine and Health Sciences, Atmajaya Catholic University of Indonesia, Jakarta, Indonesia.; ^4^Department of Child Health, Faculty of Medicine, Public Health and Nursing, Universitas Gadjah Mada, D.I.Y Jogjakarta, Indonesia.; ^5^Graduate Program of Clinical Medicine Science, Faculty of Medicine, Public Health and Nursing, Universitas Gadjah Mada, D.I.Y Jogjakarta, Indonesia.; ^6^Department of Obstetrics and Gynaecology, Faculty of Medicine Universitas Kristen Indonesia, Jakarta, Indonesia.; ^7^Diagnos Genomics, Jakarta, Indonesia.; ^8^Department of Anatomy, Physiology and Pharmacology, IPB University, Bogor, Indonesia.

**Keywords:** In-vitro fertilization, Spinal muscular atrophy, Preimplantation diagnosis.

## Abstract

**Background:**

Spinal muscular atrophy (SMA) is characterized by the homozygous deletion of the survival motor neuron-1 gene. Pre-implantation genetic testing for monogenic diseases through in-vitrofertilization program was developed to provide a reliable genetic diagnostic method for SMA.

**Case presentation:**

The couple who was confirmed as carriers of SMA visited the Morula IVF Clinic, Jakarta, Indenesia seeking for an in-vitro fertilization expert opinion in relation to the pre-implantation genetic testing for SMA. Utilizing polymerase chain reaction-restriction fragment length polymorphism, we have successfully screened for unaffected embryos that were characterized by a normal presence of the survival motor neuron-1 exon 7-8 and survival motor neuron-2 exon 7-8. The frozen embryo was subsequently transferred and a healthy unaffected female baby was born with undetected deletion of the survival motor neuron-1 gene.

**Conclusion:**

This successful embryo pre-implantation screening case could potentially accommodate the demands of genetically at-risk couples who are apprehensive about conceiving a child who might inherit monogenic disorders such as SMA.

## 1. Introduction

Spinal muscular atrophy (SMA) is a monogenic neuromuscular disorder that is estimated to occur in 1 per 10.000 live births with 1 in 50 people being the genetic carrier of the disease worldwide. SMA is a homozygous autosomal recessive disorder caused by the inactivation of the survival alpha motor neuron-1 (*SMN1*) gene caused by the gene's deletion or point mutation. In 95% of the cases, SMA-affected people present homozygous deletion of SMN-1 exon 7, while approximately 5% carry deleterious variants or point mutations of the gene.

The genetic alteration ultimately leads to a diminished expression of the survival motor neuron protein that is crucial in maintaining healthy functional motor neuron cells within the spinal cord. Inadequate expression of SMN protein compromises the survival and ability of the spinal motor neuron cells to transmit neural signals. Consequently, symptoms of SMA would include proximal muscle weakness, muscular atrophy, or hypotonia, which manifest mostly in the lower extremities (1).

*SMN-1* and survival alpha motor neuron-2 (*SMN2*) genes share a considerably similar structure. The diploid nature of the human genome implies that a normal individual carries 2 copies of both the *SMN1* and *SMN2* genes. *SMN2* gene also produces the SMN protein, but during its transcription, a nucleotide substitution at the 5' end of exon 7 generates unstable and subsequently degraded protein transcripts. Therefore, the *SMN2* gene contributes to only a limited production of functional SMN proteins. While the inactivation of the *SMN1* gene is the main cause of inherited SMA, the gene copy number of *SMN2* could determine the severity of the disease in which a higher *SMN2* gene copy number in affected people has been associated with less severe SMA symptoms (1).

Implementation of pre-implantation genetic testing for monogenic diseases (PGT-M) through in vitro fertilization (IVF) has become a promising conception strategy for couples who are at risk of passing on an abnormal gene to their child. Following procedures of IVF such as ovarian stimulation, ovum pick-up, insemination, and embryo culture, the screening of embryos for genetic disorders can be performed to permit the transfer of genetically unaffected embryos. According to a survey study conducted at 137 IVF clinics in the United States, 82% of these clinics have offered pre-implantation genetic diagnosis for single-gene disorders (2). Furthermore, the European Society Human Reproduction and Endocrinology PGT consortium data collection XVI-XVIII reported that, in 2015, approximately 2.661 cycles of IVF-PGT-M were conducted, in which 50% were utilized to detect autosomal dominant diseases while 26% were performed to detect autosomal recessive diseases including SMA (3).

Hereby, we report the first successful birth of a healthy baby from parents who are both carriers of SMA following a combination of IVF and PGT-M program at our IVF Clinic Center.

## 2. Case presentation

### Couple history

The couple visited Morula IVF Jakarta Clinic, Jakarta, Indonesia in October 2019 to seek an IVF expert opinion regarding PGT-M for SMA. The wife was 26 while the husband was 32 yr. Both had a normal karyotype (46XX and 46XY, respectively). Additionally, both spouses were related as cousins. A passing of a cousin was because of SMA was recorded in the husband's family history. Previously, the couple had their 1 ${}^{\text{st}}$ female baby who was diagnosed with SMA at the age of 7 months and passed away at the age of 15 months due to hypotonia and respiratory distress. SMA screening analysis of the affected 1 ${}^{\text{st}}$ -born exhibited the deletion of the *SMN-1* gene and the presence of 2 *SMN-2* gene copies. To avoid having another child who is affected with SMA, the couple decided to consider IVF-PGT-M.

### IVF and embryo biopsy

A complete hormonal basal profile measurements of the wife presented normal values (follicle-stimulating hormone; 6.71 mIU/mL, estradiol: 22.94 pg/mL, progesterone: 0.10 ng/mL, luteinizing hormone: 4.5 mIU/mL, and anti-Mullerian hormone: 2.73 ng/mL). The detected antral follicle count was 15. The wife was then subjected to a gonadotropin-releasing hormone antagonist protocol. The initial dose of gonadotropin (Gonal F, Merck Serono, Switzerland) was 225 IU which was not altered for 9 days with a total dose of 2025 IU. Daily priming injections of 0.25 mg Cetrotide (Merck KGaA, Darmstadt, Germany) started on day 6 of the menstrual cycle. Maturation trigger injection of 0.3 cc Buserelin (Suprefact, Sanofi-Aventis, Germany) was given 36 hrs before the ovum pick-up procedure. Buserelin was administered to prevent ovarian hyperstimulation syndrome caused by the high level of estradiol on trigger day (3115 pg/mL).

A total of 18 mature oocytes were retrieved and each was injected with a single spermatozoon from the husband through intracytoplasmic morphologically selected sperm injection. Thirteen oocytes were successfully fertilized and were subsequently cultured until day 5 after the injection procedure. On day 4, all developed embryos were subjected to a series of 3 4-μm diameter laser pulses (OCTAX Laser Shoot-TM) to facilitate trophectoderm (TE) herniation. On day 5, 3 top-quality blastocysts were then biopsied for the PGT-M process. During the biopsy process, protruded TE cells were lasered at the junction by a maximum of 3 pulses followed by aspiration into the biopsy pipette (Blastomere aspiration pipette; COOK Ireland, Ltd). Approximately 2-4 TE cells were eventually collected in 0.2 mL micro-centrifuge tubes subsequent to washing in sterile phosphate-buffered saline. The embryos were then vitrified and stored in liquid nitrogen.

### PGT-A and PGT-M

**Figure 1:**
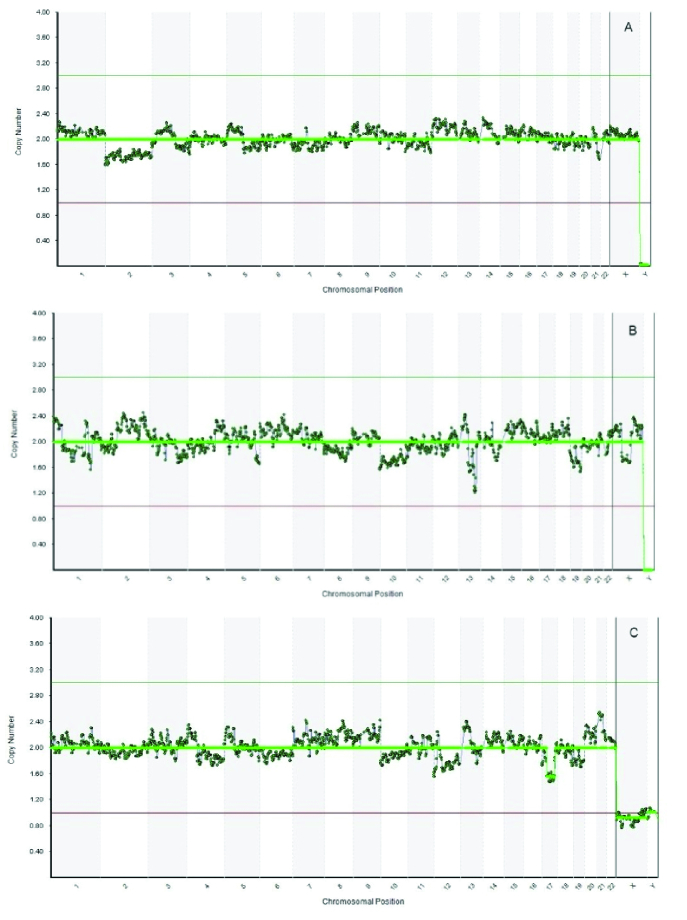
Preimplantation genetic testing for aneuplody results of the 3 top-quality embryos, (a) embryo number 1 (euploid, 46XX), (b) embryo number 2 (euploid, 46XX), (c) embryo number 3 (46XY; deletion (17q11.2-17q24.2, 39.50Mbp).

Polymerase chain reaction (PCR)-based method of whole-genome amplification (WGA) was conducted on the DNA extracted from the biopsied cells by utilizing the SurePlex DNA Amplification system (Illumina, San Diego CA). Next-generation sequencing procedure using a Veriseq PGS-MiSeq kit (Illumina, USA) was utilized for the ploidy screening. Data was interpreted using the BlueFuse Multi Software V4.5 (32178) (Illumina, USA). Eventually, 2 embryos were found to be euploid (both of which were 46XX) while the remaining one was euploid with mild mosaicism (46XY; deletion (17q11.2-17q24.2, 39.50Mbp) (Figure 1).

**Figure 2:**
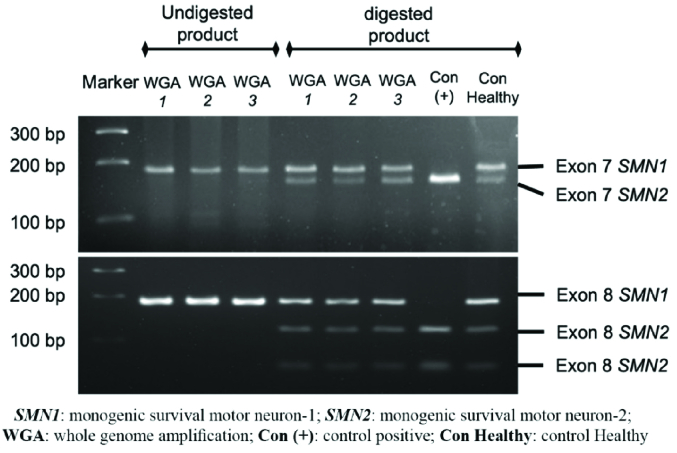
PGT-M *SMN1* deletion test results of the 3 top-quality embryos, WGA1, WGA2 and WGA3 which represented 3-top quality embryos 1, 2 and 3. 3 most left lane were undigested products of polymerase chain reaction amplification (A) SMN exon-7 amplification (187 bp), (B) SMN exon-8 amplification (186 bp). Following next wells were filled with the digested product from each embryo. The 2 most right lane were filled with samples from spinal muscular atrophy control [Con (+)] and healthy control patients [Con Healthy].

PGT-M for SMA was subsequently performed at the Genetics Laboratory of Universitas Gadjah Mada, Faculty of Medicine, Public Health and Nursing. WGA DNA samples of the embryos were tested for *SMN1* and *SMN2* exon 7-8 deletion using the PCR-restriction fragment length polymorphism (RFLP) method. Previously described primer sequences were designed to amplify the allele-specific *SMN-1* and *SMN-2* genes (Table I) (4, 5). The resulting PCR products were subsequently digested with restriction enzymes *Dra*I and *Dde*I to isolate the DNA sequence of interest: exon 7 and exon 8, respectively. The digested DNA was then gel-electrophorized in 4% agarose and 3% agarose for exon 7 and exon 8, respectively. The DNA bands were then identified under ultra-violet light. The expected bands for *SMN* exon 7 and exon 8 after PCR were 187 bp and 186 bp. After enzymatic digestion, the *SMN *exon 7 band in the healthy embrio was expected to be cleaved into 2 bands, while exon 8 was cleaved into 3 bands, as shown in figure 2. SMA positive and negative control samples were provided for comparison and quality control of the PCR and digestion process. Here, *SMN1* deletion was confirmed when the digestion only resulted in 1 band of *SMN* exon 7 and 2 bands of *SMN exon 8*.

The results indicated that all the embryos possessed the full-length functional sequence of *SMN1* and *SMN2* exon 7-8, implying that the embryos were not affected by the *SMN1*-deletion type SMA.

### Frozen embryo transfer

After genetic counseling and discussion, the couple eventually opted to undergo a frozen embryo transfer of one of the euploid embryos (number 1) through a natural cycle. The embryo survived the thawing process and was successfully transferred into the mother's uterus without any difficulties. Luteal support using 200 mg Utrogestan (Bezins-Belgium) was vaginally administered every 12 hr starting on the day of the embryo transfer until the beta human chorionic gonadotropin test day. Chemically tested beta human chorionic gonadotropin level was 6904.00 mIU/mL suggesting successful implantation of the embryo. Fetal growth was frequently monitored. There were no complications during the pregnancy and a healthy female baby was born at 37 wk and 6 days. Overall clinical presentation of the born baby was favorable. The weight, body length and head circumference at birth were 3020 gr, 49 cm, and 34 cm, respectively, with an Apgar score of 9/10. The baby had normal active movements, normal muscle tones, and suckling reflex. Genetic analysis of the newborn baby was carried out 21 days after birth to identify *SMN1* and *SMN2* exon 7-8 deletion. The PCR - RFLP results showed presence of the *SMN1* exon 7-8 and *SMN2* exon 7-8 which implied that the baby was not affected with the congenital SMA.

### Ethical considerations

A signed informed consent was obtained from the couple for the publication of this study. The case protocol was approved by the Faculty of Medicine, University of Indonesia Ethics Committee, Jakarta, Indonesia (Code: ND-120/UN2.F1/ETIK/PPM.00.02/2021).

**Table 1:** Primer design for polimerase chain reaction


**Primer for ** ***SMN*** ** gene**		**Sequence**
***SMN*** ** exon-7**		
	**Forward**	5'-AGACTATCAACTTAATTTCTGATCA-3'
	**Reverse**	5'-CCTTCCTTCTTTTTGATTTTGTTT-3'
***SMN*** ** exon-8**		
	**Forward**	5'-GTAATAACCAAATGCAATGTGAA-3'
	**Reverse**	5'-CTACAACACCCTTCTCACAG-3'
*SMN*: Survival motor neuron		

## 3. Discussion

This study presented the usefulness of the PGT-A-PGT-M service in a private IVF clinic as a screening strategy to prevent conceiving a baby who would inherit SMA disorder from parents with SMA carrier status (heterozygous deletion of *SMN1*). The IVF-PGT-M procedures allow early detection of the disease at the pre-implantation blastocysts stage by utilizing minimal biopsy of the TE cells. Although IVF is complex and costly, the couple was driven to go through with the program to avoid the mental anguish of overcoming possible pregnancy terminations and having another child who would suffer from the same life-threatening conditions. Geneticists and IVF specialists also took an important role during the counselling in which clear and comprehensive information concerning the possible risks and advantages of the program was given to the couple. An increasing trend of genetically at-risk couples considering PGT-M in their attempt to conceive through IVF is observed in several studies (6, 7). According to a survey study, the supply of clear information on the accuracy of PGT-M served as one of the most important factors that determined the couples' decision to go through with the IVF/PGT-M procedure (6).

The relatively high carrier frequency of SMA has demanded the development of a reliable molecular diagnostic technique, particularly in Indonesia, to accommodate the specific needs of couples who are at risk of passing on the genetic condition to their child. Multiple genetic testing modalities for SMA have long been developed such as sequencing, quantitative PCR (qPCR), PCR- RLFP. Each of these methods, however, has its limitations and advantages (8). Notably, one of the main challenges of performing PGT-M is the limited amount of genetic sample volume for the analysis. Multiple displacement amplification was therefore suggested as a promising technique for the amplification of single or few cell samples (7).

Detection of *SMN1* deletion combined with several marker-based linkage analyses that have a high predictive value for heterozygosity have also been demonstrated (9). In this case, we utilized PCR to amplify exon 7-8 of SMN followed by RFLP employing *Dra*I and *Dde*I enzymes to identify the *SMN1* gene exon 7-8 deletion. This method has a limitation in which the copy number of *SMN1* and *SMN2* genes could not be examined; thus, the SMA carrier status of the baby could not be confirmed. Additional diagnostic procedures such as quantitative PCR are essential to obtain comprehensive results. Identification of *SMN1* gene exon 7 and the gene copy number could be achieved by combining PCR-RFLP with multiplex ligation-dependent probe amplification method (5, 10). However, to date, several studies have reported the effectiveness of a modified nested PCR-RFLP in depicting the copy number of the *SMN1* gene.

Despite limitation, the advantages of using PCR-RFLP method for PGT-M are quite apparent, such as: 1) inter-laboratory reproducibility, 2) low cost, 3) utilization of simple standard equipment, 4) straightforward qualitative analysis without the need for a quantification procedure to determine the positive status, 5) yield of robust results while utilizing a small quantity of DNA derived from an embryo biopsy sample. Additionally, given that 95% of people who are clinically diagnosed with SMA have the undetectable exon 7 and 8 of *SMN1*, this method permits an efficient early diagnosis for SMA (10).

Although most embryos biopsy for PGT-M were executed at the cleavage stage (3), we believe that performing the biopsy on the blastocyst stage is more convenient, especially for developing countries such as Indonesia. Considering the great expense of IVF treatments along with the additional costs of PGT-A and PGT-M, conducting a genetic test at the cleavage-stage is less appropriate as the development of the embryos up to the blastocyst stage would not be guaranteed; patients could end up with blastocysts that are not suitable for transfer thus the outcomes of prior genetic test at the cleavage stage would be futile.

## 4. Conclusion

The success of our 1 ${}^{\text{st}}$ IVF-PGT-M case has proven the effectiveness of the pre-implantation molecular diagnosis of SMA. Although other diagnostic tools are needed to confirm the copy number of *SMN* genes, detection of *SMN1* gene deletion is feasible through the RFLP-PCR method.

##  Conflict of Interest

The authors declared that there is no conflict of interest.
